# Molecular Epidemiology and Drug Resistant Mechanism of Carbapenem-Resistant *Klebsiella pneumoniae* in Elderly Patients With Lower Respiratory Tract Infection

**DOI:** 10.3389/fpubh.2021.669173

**Published:** 2021-05-20

**Authors:** Chunhong Shao, Wei Wang, Shuang Liu, Zhijun Zhang, Meijie Jiang, Fusen Zhang

**Affiliations:** ^1^Clinical Laboratory of Shandong Provincial Hospital Affiliated to Shandong First Medical University, Shandong, China; ^2^Intensive Care Department of Taian City Central Hospital, Shandong, China; ^3^Hematology Department of Taian City Central Hospital, Shandong, China; ^4^Clinical Laboratory of Taian City Central Hospital, Shandong, China

**Keywords:** *K. pneumoniae*, carbapenemase, elderly, drug resistance, lower respiratory tract infection

## Abstract

Infection by carbapenem-resistant *Klebsiella pneumoniae* (CRKp) hampers the treatment of elderly patients with lower respiratory tract infection (LRTI); however, relevant data with respect to the characteristics of CRKp in elderly patients with LRTIs are limited. In the present study, *K. pneumoniae* isolated from elderly patients with LRTIs was collected and identified by VITEK-MS. VITEK 2 compact was used for drug sensitivity test to screen CRKps, and broth dilution method was used for drug sensitivity of tigecycline and colistin. The resistance genes, virulence genes, and serotypes of CRKps were detected via polymerase chain reaction. The homology of CRKps was analyzed via PFGE and MLST. Moreover, plasmid conjugation experiment was carried out to determine the transferability of carbapenem resistance. PCR-based replicon typing (PBRT) and S1 nuclease-PFGE were conducted for plasmid profiling. From January 2019 to August 2019, 258 elderly patients with LRTIs caused by *K. pneumoniae* were observed; of these, 31 (12.02%) infections were caused by CRKp strains. Majority of the patients were admitted to the intensive care unit and neurosurgery wards. Intracranial hemorrhage and pneumonia were the most common underlying diseases. Furthermore, 29 patients infected by CRKp had been exposed to various antimicrobial drugs before the positive culture. All isolates exhibited high resistance to β-lactam antibiotics. The predominant carbapenem resistance gene was *bla*_KPC−2_, and CRKps carrying *bla*_KPC−2_ were all ST11 type. Two *bla*_NDM−5_ carrying isolates were assigned to ST307 and ST1562, respectively. Conjugative assays revealed that plasmids harboring *bla*_NDM−5_ gene were self-transmissible. Plasmid analysis suggested that two *bla*_NDM−5_ were located on a ~45 kb IncX3 type plasmid. The high incidence of CRKp in elderly patients with LRTIs indicates the urgent need for further surveillance and strict infection control measures.

## Introduction

Carbapenem-resistant *Enterobacteriaceae* (CRE) presents an urgent public health concern worldwide due to rapidly rising resistance rates and subsequent high mortality. Report from China CRE Network revealed that most CRE cases were caused by *Klebsiella pneumoniae* (73.9%) (1). *K. pneumoniae* is a common causative pathogen of various nosocomial infections, including pneumonia, urinary tract infection, abdominal infection, and bacteremia. Despite improvements in hospital infection control and antimicrobial scientific stewardship, carbapenem-resistant *K. pneumoniae* (CRKp) is still on the rise (2, 3). Surveillance of antibiotic resistance by CHINET in China revealed that 3.0 and 2.9% of *Klebsiella spp*. were resistant to imipenem and meropenem, respectively, in 2005, compared to 25.3 and 26.8%, respectively, in 2019 (http://chinets.com/Data/GermYear). This antibiotic resistance poses a greater challenge in clinical treatment of infection caused by CRKp.

Lower respiratory tract infections (LRTIs) are a leading cause of mortality and morbidity worldwide (4). Community- or hospital-acquired LRTIs are highly prevalent in the elderly. In developing countries, the situation is more complicated, and management is often difficult due to the identification of etiological agents and administration of an appropriate treatment in cases requiring antibiotic therapy. *K. pneumoniae* is an important pathogen causing LRTIs in the elderly (5). A report from China CRE Network revealed that 65.4% of the CRE patients presented LRTIs, and it is more serious in the elderly (1). Infection by CRKp makes the treatment of elderly patients with LRTIs face greater challenges.

Although CRKp has led to wide global disseminations and serious clinical outcomes, limited data is available on the molecular epidemiology of this pathogen in elderly patients with LRTIs. Therefore, we conducted this study to investigate the resistance profiles, molecular epidemiology, and clinical characteristics of CRKp isolates obtained from elderly patients with LRTIs.

## Materials and Methods

### Bacterial Isolates and Patients

*K. pneumoniae* strains were collected from lower respiratory tract specimens, including bronchoalveolar lavage fluid, sputum, and pleural effusion, obtained from individual elderly patients (age ≥ 60 years) admitted to Taian City Central Hospital in China from January 2019 to August 2019. Elderly patients who presented at least two of the following symptoms were included in the study: fever, cough, dyspnoea, wheezing, chest pain, or sore throat. Any prior antimicrobial treatment taken by the patient was also recorded before microbiological investigations. Patients diagnosed with pulmonary tuberculosis or with infections other than LRTIs were excluded. Patient information including age, gender, diagnosis, treatment, and outcomes was obtained from the Electronic Medical Records. The methods in this study were approved by the Ethics Committee of Taian City Central Hospital and were carried out in accordance with the approved guidelines. The VITEK-MS (bioMérieux, France) was used to identify the bacterial strains. CRKp was defined as the minimal inhibition concentration (MIC) of ertapenem ≥2 μg/mL or the MIC of imipenem and meropenem ≥ 4 μg/mL (6). Only the first episode of CRKp-associated LRTIs was included.

### Antimicrobial Susceptibility Testing

Antimicrobial susceptibility testing was performed by VITEK 2 compact system using GN13 cards (bioMérieux, France) according to the manufacturer's instructions. The MICs of imipenem, meropenem, and ertapenem were determined by an *E*-test (bioMérieux); whereas, MICs of tigecycline and colistin were determined by broth microdilution method (Bio-kont, China). *Escherichia coli* ATCC25922 and *K. pneumoniae* ATCC700603 (American Type Culture Collection Center, Manassas, VA, USA) acted as the quality controls. All antibiotics were administered according to the 2019 European Committee on Antimicrobial Susceptibility Testing breakpoint (www.eucast.org/clinical_breakpoint). Susceptibility data were analyzed using WHONET 5.6 software recommended by the World Health Organization.

### PCR and DNA Sequence Analysis of Drug Resistance Genes, Serotype, and Virulence Genes

Samples were screened for the presence of carbapenem resistance genes (*bla*_KPC_, *bla*_SME_, *bla*_IMI_, *bla*_NMC_, *bla*_GES_, *bla*_IMP_, *bla*_VIM_, *bla*_GIM_, *bla*_SIM_, *bla*_SPM_, *bla*_NDM_, and *bla*_OXA−48like_), other β-lactamase genes (*bla*_CTX−M_, *bla*_TEM_, *bla*_SHV_, *bla*_MOX_, *bla*_FOX_, *bla*_DHA_, *bla*_CIT_, *and bla*_EBC_), and integron structures (Int1, Int2, Int3) (7, 8). For CRKp isolates that were resistant to quinolones or aminoglycoside, plasmid-mediated quinolone resistance genes [*qnrA, qnrB, qnrC, qnrD, qnrS, qepA, and aac(6)-Ib-cr*] or 16S rRNA methyase gene *(rmtA, rmtB, rmtC, rmtD, npmA*, and *armA*) were screened (9, 10). Primers used in the present study were listed in Supplementary Table 1.

The isolates were serotyped for serotypes K1, K2, K5, K20, K54, and K57, and moreover, 12 virulence-associated genes, including *rmpA, aerobactin, wcaG, ybtA, iucB, iroNB, ureA, uge, kfuBC, fim, wabG*, and *allS*, were screened using PCR as previously reported (11). Nucleotide sequences were analyzed and compared using BLAST (http://www.ncbi.nlm.nih.gov/blast).

### Pulse-Field Gel Electrophoresis

An overnight bacterial culture was suspended in cell suspension buffer [100 mM EDTA, 100 mM Tris-HCl (pH 8.0)] and adjusted to an optical density of 4.0 at a wave length of 600 nm. The suspension was mixed with equal volumes 2% solution of low-melting agarose in Tris-EDTA [TE: 1 mM EDTA, 10 mM Tris-HCl (pH 8.0)]. After cooling, the agarose sections were incubated for 4 h at 54°C in cell lysis buffer [50 mM Tris-HCl, 50 mM EDTA (pH 8.0), 0.01 g/ml N-lauroyl-sarcosine, sodium salt, 0.1 mg/ml proteinase K]. Thereafter, the sections were washed thoroughly with TE buffer and digested overnight with XbaI restriction endonuclease (Takara Bio, Inc., Otsu, Japan). Genomic DNA was separated in 0.5× Tris/borate/EDTA (TBE) buffer in a PFGE system (CHEF Mapper; Bio-Rad Laboratories, Inc., Hercules, CA, USA) at 14°C, using a voltage of 6 V/cm, a switch angle of 120°, and a switch ramp of 6–36 s for 21 h.

### Multilocus Sequence Typing

MLST of *K. pneumoniae* was performed according to protocols available on the MLST Pasteur website (http://www.pasteur.fr/recherche/genopole/PF8/mlst/Kpneumoniae.html). Seven conserved housekeeping genes (*gapA, infB, mdh, pgi, phoE, rpoB*, and *tonB*) were amplified, sequenced, and compared with those in the MLST databases.

### Conjugation Assay and Analysis of Plasmids

Conjugation was performed using the mixed broth method. Briefly, *E. coli J53* Azi^R^ was used as the recipient strain and 31 clinical CRKps served as the donors. Transconjugants were selected on Mueller Hinton agar supplemented with meropenem (0.5 μg/mL) and sodium azide (100 μg/mL). The transconjugants were identified by VITEK-MS. Antimicrobial susceptibility test of the transconjugant was carried out as described for the clinical strain.

Plasmid incompatibility types of the transconjugants were identified via PCR-based replicon typing, as reported previously (8). The size and amount of plasmids carried by the 31 clinical isolates and transconjugants were evaluated using S1-pulsed-field gel electrophoresis (S1-PFGE), as previously described (12). The genome of *Salmonella* H9812 digested with XbaI was used as the marker.

## Results

### Clinical Characteristics of Patients With LRTIs Caused by CRKp

From January 2019 to August 2019, 258 elderly patients with LRTIs caused by *K. pneumoniae* were observed; of these, 31 (12.02%) were caused by CRKp strains. The median age of 31 patients was 75.38 years (range: 60–92 years), and 58.06% (18/31) were male patients. Moreover, 13 patients (41.94 %) were hospitalized in the intensive care unit, and 9 patients in the neurosurgery ward. Intracranial hemorrhage and pneumonia were the most common underlying diseases. Furthermore, 22 (70.97%) patients received endotracheal intubation. The baseline clinical characteristics of the patients are presented in Table 1.

**Table 1:** Clinical characteristics of 31 elderly patients with LRTIs by CRKp.

**No**.	**Gender**	**Age**	**Ward**	**Clinical diagnosis**	**Specimen**	**Antibiotic therapy(Before culture)**	**Antibiotic therapy(After culture)**	**Admission date**	**Isolate date**	**Discharge date**	**Endotracheal intubation**	**Prognosis**
P1	F	86	ICU	Severe pneumonia	Pleural effusion	MEM, LZD, SCF, TZP, MFX	TGC	2019/1/24	2019/2/15	2019/3/15	Yes	Automatic discharge
P2	F	66	General Medical Ward	Multiple system atrophy	BALF	TZP, AK	AK	2019/1/3	2019/2/16	2019/3/10	Yes	Automatic discharge
P3	F	85	Respiratory	Pneumonia	BALF	VAN, SCF		2019/2/11	2019/2/19	2019/3/5	Yes	Automatic discharge
P4	M	81	Cardic department	Heart failure	Sputum	TZP, LEV, IMP	TGC	2019/3/1	2019/3/19	2019/3/27	No	Got better
P5	M	77	EICU	COPD	Sputum	TZP, SCF	TGC	2019/3/7	2019/3/18	2019/3/28	Yes	Got better
P6	F	60	Neurosurgery	Intracranial hemorrhage	Sputum			2019/4/4	2019/4/11	2019/4/22	Yes	Transfered
P7	M	68	Neurosurgery	Intracranial hemorrhage	Sputum	TZP	TGC	2019/4/3	2019/4/11	2019/4/24	Yes	Got better
P8	F	69	Neurosurgery	Intracranial hemorrhage	Sputum	TZP	TGC	2019/4/9	2019/4/18	2019/4/28	Yes	Got better
P9	F	85	Respiratory	Pneumonia	BALF	MEM		2019/2/26	2019/4/24	2019/5/8	Yes	Transfered
P10	M	82	ICU	Fever	BALF	MEM, VRC, TZP		2019/4/4	2019/4/23	2019/5/8	No	Got better
P11	M	61	Nephrology	chronic renal failure	BALF			2019/2/12	2019/4/28	2019/5/4	No	Got better
P12	F	62	Respiratory	COPD and pneumonia	Sputum	SCF, IMP, TGC, VRC	TGC	2019/4/21	2019/4/28	2019/5/2	No	Got better
P13	M	65	Neurosurgery	Intracranial injury	Sputum	TZP, TGC	TGC	2019/4/24	2019/4/29	2019/5/19	Yes	Got better
P14	M	76	Neurosurgery	Intracranial hemorrhage	Sputum	TZP, FOX		2019/5/2	2019/5/11	1905/7/11	Yes	Got better
P15	F	61	Neurosurgery	Intracranial hemorrhage	BALF	TZP	TGC	2019/4/27	2019/5/11	2019/5/25	Yes	Got better
P16	F	82	Neurosurgery	Intracranial hemorrhage	Sputum	SCF, TZP		2019/5/5	2019/5/19	2019/5/24	Yes	Transfered
P17	F	71	ICU	Jaundice	Pleural effusion	TZP, LZD		2019/7/11	2019/8/6	2019/8/7	Yes	Automatic discharge
P18	M	79	ICU	Cardiac insufficiency	Pleural effusion	MEM, LEV, IMP	TGC	2019/1/25	2019/5/25	2019/6/14	Yes	Automatic discharge
P19	F	92	ICU	Epilepsy	Sputum	LEV, MEM, LZD	AK	2019/5/10	2019/5/26	2019/6/12	Yes	Got better
P20	M	79	ICU	Bellyache	Sputum	MEM		2019/5/29	2019/5/30	2019/6/10	No	Got better
P21	M	76	ICU	Abdominal distention	Sputum	SCF, VRC	TGC	2019/5/20	2019/6/10	2019/6/19	No	Automatic discharge
P22	M	84	Nephrology	chronic renal failure	BALF	TZP, LZD, CAZ		2019/5/15	2019/6/12	2019/6/27	No	Automatic discharge
P23	M	74	ICU	Cerebral vascular disease	Sputum	CAZ, MEM, LEV, SCF, LZD, TGC	TGC	2019/5/8	2019/6/13	2019/8/22	Yes	Got better
P24	M	91	ICU	Intracranial injury	Sputum	CAZ, TZP	TGC	2019/6/6	2019/6/21	2019/12/30	Yes	Death
P25	M	64	ICU	Shock	Sputum	MEM, TZP, AK	AK	2019/6/17	2019/6/26	2019/8/9	No	Got better
P26	F	87	Respiratory	Pneumonia	BALF	TZP, SCF	TGC	2019/4/23	2019/7/1	2019/7/15	Yes	Automatic discharge
P27	M	84	ICU	Jaundice	BALF	TZP, IMP, FEP, LEV	LEV	2019/6/5	2019/7/8	2019/7/15	Yes	Automatic discharge
P28	M	91	ICU	Intracranial injury	BALF			2019/6/27	2019/7/11	2019/8/9	Yes	Transfered
P29	F	70	Neurosurgery	Intracranial injury	Sputum	TZP	TGC	2019/3/30	2019/4/6	2019/4/23	Yes	Got better
P30	M	68	Neurosurgery	Intracranial hemorrhage	Sputum	CAZ, LEV, TZP	TGC	2019/7/25	2019/8/4	2019/9/17	Yes	Got better
P31	M	61	Nephrology	Chronic renal failure	Sputum	TZP, LZD		2019/4/10	2019/4/13	2019/5/7	No	Automatic discharge

*F, female; M, man; N, no; Y, yes; ICU, intensive care unit; AK, amikacin; CAZ, ceftazidime; FEP, cefepime; FOX, cefoxitin; IMP, imipenem; LEV, levofloxacin; LZD, linezolid; MEM, meropenem; MFX, moxifloxacin; SCF, cefoperazone/sulbactam; TGC, tigecycline; TZP, piperacillin-tazobactam; VAN, vancomycin; VRC, voriconazole*.

Additionally, 29 patients were exposed to various antimicrobial drugs before the positive culture (Table 1). The main antibiotics used included β-lactam/β-lactamase inhibitor combinations, cephalosporins, carbapenems, quinolones, vancomycin, and linezolid. Based on *in vitro* susceptibility testing results, 80.65% (25/31) of the patients received inappropriate empirical treatment. Of these, 41.94% (13/31) switched to appropriate drugs such as tigecycline, levofloxacin, and amikacin after susceptibility results were available. Antimicrobial treatment adjustment and outcomes are presented in Table 1. Eventually, 16 patients improved, 14 patients were discharged automatically or transferred to other hospitals, and only one patient died.

### Susceptibility Results of CRKp Isolates and Drug Resistance Genes

The antimicrobial susceptibility profiles of the CRKp isolates are listed in Table 2. Only Kp22 was sensitive to aztreonam. Other isolates exhibited high resistance to cephalosporin, β-lactam/β-lactamase inhibitor combinations, and carbapenems. Kp27 was sensitive to levofloxacin and ciprofloxacin. The resistance rate of 31 isolates to gentamicin and amikacin was 19.35%. Among the 31 isolates, 27 strains (87.10%) were susceptible to trimethoprim-sulfamethoxazole and all isolates were susceptible to colistin and tigecycline (100%).

**Table 2:** The MIC of 31 CRKp isolates and two transconjugants (μg/mL).

**No**.	**SAM**	**TZP**	**ATM**	**CRO**	**CAZ**	**FEP**	**FOX**	**ETP**	**IMP**	**MEM**	**SXT**	**CIP**	**LEV**	**CN**	**AK**	**TGC**	**CO**
Kp1	>16/8	≥128	>16	≥64	≥64	≥64	>16	>32	>32	>32	≥16/304	≥4	≥8	≤ 1	≤ 2	1	0.25
Kp2	>16/8	≥128	>16	≥64	≥64	≥64	>16	>32	>32	>32	≤ 1/19	≥4	≥8	≤ 1	≤ 2	0.5	0.25
Kp3	>16/8	≥128	>16	≥64	≥64	≥64	>16	>32	>32	>32	≤ 1/19	≥4	≥8	≥16	≥64	0.25	1
Kp4	>16/8	≥128	>16	≥64	≥64	≥64	>16	>32	>32	>32	≥16/304	≥4	≥8	≥16	≥64	0.5	0.5
Kp5	>16/8	≥128	>16	≥64	≥64	≥64	>16	>32	>32	>32	≤ 1/19	≥4	≥8	≥16	≥64	0.5	0.5
Kp6	>16/8	≥128	>16	≥64	≥64	≥64	>16	>32	>32	>32	≤ 1/19	≥4	≥8	≥16	≥64	0.25	0.5
Kp7	>16/8	≥128	>16	≥64	≥64	≥64	>16	>32	>32	>32	≤ 1/19	≥4	≥8	≥16	≥64	0.5	0.5
Kp8	>16/8	≥128	>16	≥64	≥64	≥64	>16	>32	>32	>32	≤ 1/19	≥4	≥8	≥16	≥64	1	0.5
Kp9	>16/8	≥128	>16	≥64	≥64	≥64	>16	>32	>32	>32	≤ 1/19	≥4	≥8	≥16	≥64	1	1
Kp10	>16/8	≥128	>16	≥64	≥64	≥64	>16	>32	>32	>32	≥16/304	≥4	≥8	≥16	≥64	0.25	0.5
Kp11	>16/8	≥128	>16	≥64	≥64	≥64	>16	>32	>32	>32	≤ 1/19	≥4	≥8	≥16	≥64	0.5	1
Kp12	>16/8	≥128	>16	≥64	≥64	≥64	>16	>32	>32	>32	≤ 1/19	≥4	≥8	≥16	≥64	0.5	0.25
Kp13	>16/8	≥128	>16	≥64	≥64	≥64	>16	>32	>32	>32	≤ 1/19	≥4	≥8	≥16	≥64	0.5	0.25
Kp14	>16/8	≥128	>16	≥64	≥64	≥64	>16	>32	>32	>32	≤ 1/19	≥4	≥8	≥16	≥64	0.25	0.5
Kp15	>16/8	≥128	>16	≥64	≥64	≥64	>16	>32	>32	>32	≤ 1/19	≥4	≥8	≥16	≥64	0.25	0.5
Kp16	>16/8	≥128	>16	≥64	≥64	≥64	>16	>32	>32	>32	≤ 1/19	≥4	≥8	≥16	≥64	0.25	0.5
Kp17	>16/8	≥128	>16	≥64	≥64	≥64	>16	>32	>32	>32	≤ 1/19	≥4	≥8	≥16	≥64	0.25	0.25
Kp18	>16/8	≥128	>16	≥64	≥64	≥64	>16	>32	>32	>32	≤ 1/19	≥4	≥8	≥16	≥64	0.5	0.25
Kp19	>16/8	≥128	>16	≥64	≥64	≥64	>16	>32	>32	>32	≤ 1/19	≥4	≥8	≤ 1	≤ 2	0.5	0.5
Kp20	>16/8	≥128	>16	≥64	≥64	≥64	>16	>32	>32	>32	≤ 1/19	≥4	≥8	≥16	≥64	0.25	0.5
Kp21	>16/8	≥128	>16	≥64	≥64	≥64	>16	>32	>32	>32	≤ 1/19	≥4	≥8	≤ 1	≤ 2	0.5	0.5
Kp22	>16/8	≥128	≤ 4	≥64	≥64	≥64	>16	>32	>32	>32	≤ 1/19	≥4	≥8	≥16	≥64	1	0.5
Kp23	>16/8	≥128	>16	≥64	≥64	≥64	>16	>32	>32	>32	≤ 1/19	≥4	≥8	≥16	≥64	1	0.5
Kp24	>16/8	≥128	>16	≥64	≥64	≥64	>16	>32	>32	>32	≤ 1/19	≥4	≥8	≥16	≥64	0.25	1
Kp25	>16/8	≥128	>16	≥64	≥64	≥64	>16	>32	>32	>32	≤ 1/19	≥4	≥8	≥16	≥64	0.5	0.5
Kp26	>16/8	≥128	>16	≥64	≥64	≥64	>16	>32	>32	>32	≥16/304	≥4	≥8	≥16	≥64	0.5	1
Kp27	>16/8	≥128	>16	≥64	≥64	≥64	>16	>32	>32	>32	≤ 1/19	≤ 0.25	≤ 0.25	≤ 1	≤ 2	1	0.25
Kp28	>16/8	≥128	>16	≥64	≥64	≥64	>16	>32	>32	>32	≤ 1/19	≥4	≥8	≥16	≥64	0.5	0.25
Kp29	>16/8	≥128	>16	≥64	≥64	≥64	>16	>32	>32	>32	≤ 1/19	≥4	≥8	≥16	≥64	0.25	1
Kp30	>16/8	≥128	>16	≥64	≥64	≥64	>16	>32	>32	>32	≤ 1/19	≥4	≥8	≥16	≥64	0.5	0.5
Kp31	>16/8	≥128	>16	≥64	≥64	≥64	>16	>32	>32	>32	≤ 1/19	≥4	≥8	≤ 1	≤ 2	0.5	0.5
J22	>16/8	≥128	≤ 4	≥64	≥64	≥64	>16	>32	>32	>32	≤ 1/19	≤ 0.25	≤ 0.25	≤ 1	≤ 2	0.125	0.125
J27	>16/8	≥128	≤ 4	≥64	≥64	≥64	>16	>32	>32	>32	≤ 1/19	≤ 0.25	≤ 0.25	≤ 1	≤ 2	0.125	0.125

*MIC, Minimum inhibitory concentration; SAM, ampicillin sulbactam; TZP, piperacillin/tazobactam; ATM, aztreonam; CRO, ceftriaxone; CAZ, ceftazidime; FEP, cefepime; FOX, cefoxitin; ETP, ertapenem; IPM, imipenem; MEM, meropenem; SXT, trimethoprim-sulfamethoxazole; CIP, ciprofloxacin; LEV, levofloxacin; CN, gentamicin; AK, amikacin; TGC, tigecycline; CO, colisin*.

The predominant carbapenem resistance gene was *bla*_KPC−2_ (93.55%, 29/31). Two isolates carried *bla*_NDM−5_. In addition to carbapenem resistance genes, we examined other types of β-lactamase genes, including ESBLs and AmpC genes. The distribution patterns of resistance genes in these strains are listed in Table 3. We identified *bla*_TEM−1_, *bla*_SHV−1_, and *bla*_CTX−M−1group_ in 31 (100%), 28 (90.32%), and 27 (87.10%) isolates, respectively. Moreover, 30 isolates carried class I integron. Among the 25 aminoglycoside resistant CRKps, 23 isolates carried the *rmtB* gene, and Kp23 also carried *armA*. Kp3 and Kp9 carried only *armA*.

**Table 3:** The drug-resistance genes and virulence genes of 31 CRKp isolates.

	**Drug-resistance genes**								**Virulence genes**					
	**β-lactamases**						**Aminoglycoside**		**Capsule**			**Fimbriae**		**Iron acquistion**
**No**.	***bla*_**KPC-2**_**	***bla*_**NDM-5**_**	***bla*_**SHV-11**_**	***bla*_**TEM-1**_**	***bla*_**CTX-M-1 group**_**	**Class I integrons**	**rmtB**	**armA**	**rmpA**	**wabG**	**uge**	**fimH**	**mrkD**	**iucB**
Kp1	*		*	*		*				*	*	*	*	
Kp2	*		*	*		*				*	*	*	*	
Kp3	*		*	*	*	*		*		*	*	*	*	*
Kp4	*		*	*	*	*	*			*	*	*	*	*
Kp5	*		*	*	*	*	*		*	*	*	*	*	*
Kp6	*		*	*	*	*	*		*	*	*	*	*	
Kp7	*		*	*	*	*	*		*	*	*	*	*	*
Kp8	*		*	*	*	*	*		*	*	*	*	*	*
Kp9	*		*	*	*	*		*		*	*	*	*	*
Kp10	*		*	*	*	*	*		*	*	*	*	*	*
Kp11	*		*	*	*	*	*		*	*	*	*	*	*
Kp12	*		*	*	*	*	*		*	*	*	*	*	*
Kp13	*		*	*	*	*	*		*	*	*	*	*	*
Kp14	*		*	*		*	*		*	*	*	*	*	*
Kp15	*		*	*	*	*	*		*	*	*	*	*	*
Kp16	*		*	*	*	*	*		*	*	*	*	*	*
Kp17	*		*	*	*	*	*		*	*	*	*	*	*
Kp18	*		*	*	*	*	*		*	*	*	*	*	*
Kp19	*			*	*	*				*	*	*	*	
Kp20	*		*	*	*	*	*		*	*	*	*	*	*
Kp21	*		*	*	*	*	*		*	*	*	*	*	*
Kp22		*		*			*			*	*	*	*	*
Kp23	*		*	*	*	*	*	*	*	*	*	*	*	*
Kp24	*		*	*	*	*	*		*	*	*	*	*	*
Kp25	*		*	*	*	*	*		*	*	*	*	*	*
Kp26	*		*	*	*	*	*			*	*	*	*	
Kp27		*		*	*	*		*	*	*	*	*	*	
Kp28	*		*	*	*	*	*		*	*	*	*	*	*
Kp29	*		*	*	*	*	*		*	*	*	*	*	*
Kp30	*		*	*	*	*	*		*	*	*	*	*	*
Kp31	*		*	*	*	*	*	*	*	*	*	*	*	*

^*^: positive; blank: negative.

### Serotype and Virulence Genes

The serotype and 12 virulence-associated genes of CRKp isolates were analyzed. For the six serotypes closely related to hypervirulent *K. pneumoniae*, all strains exhibited negative results. Among the 12 virulence-associated genes, *fim, uge, mrkD, and wabG* were harbored by all 31 isolates. In addition, *rmpA* and *iucB* were also harbored by 74.19% (23/31) and 80.65% (25/31) of the strains, respectively; however, the other six genes including, *aerobactin, wcaG, ybtA, iroNB, ureA, kfuBC*, and *allS* were not detected in any of the 31 isolates. The detailed results are listed in Table 3.

### PFGE and MLST Analysis of CRKp Isolates

**Figure 1:**
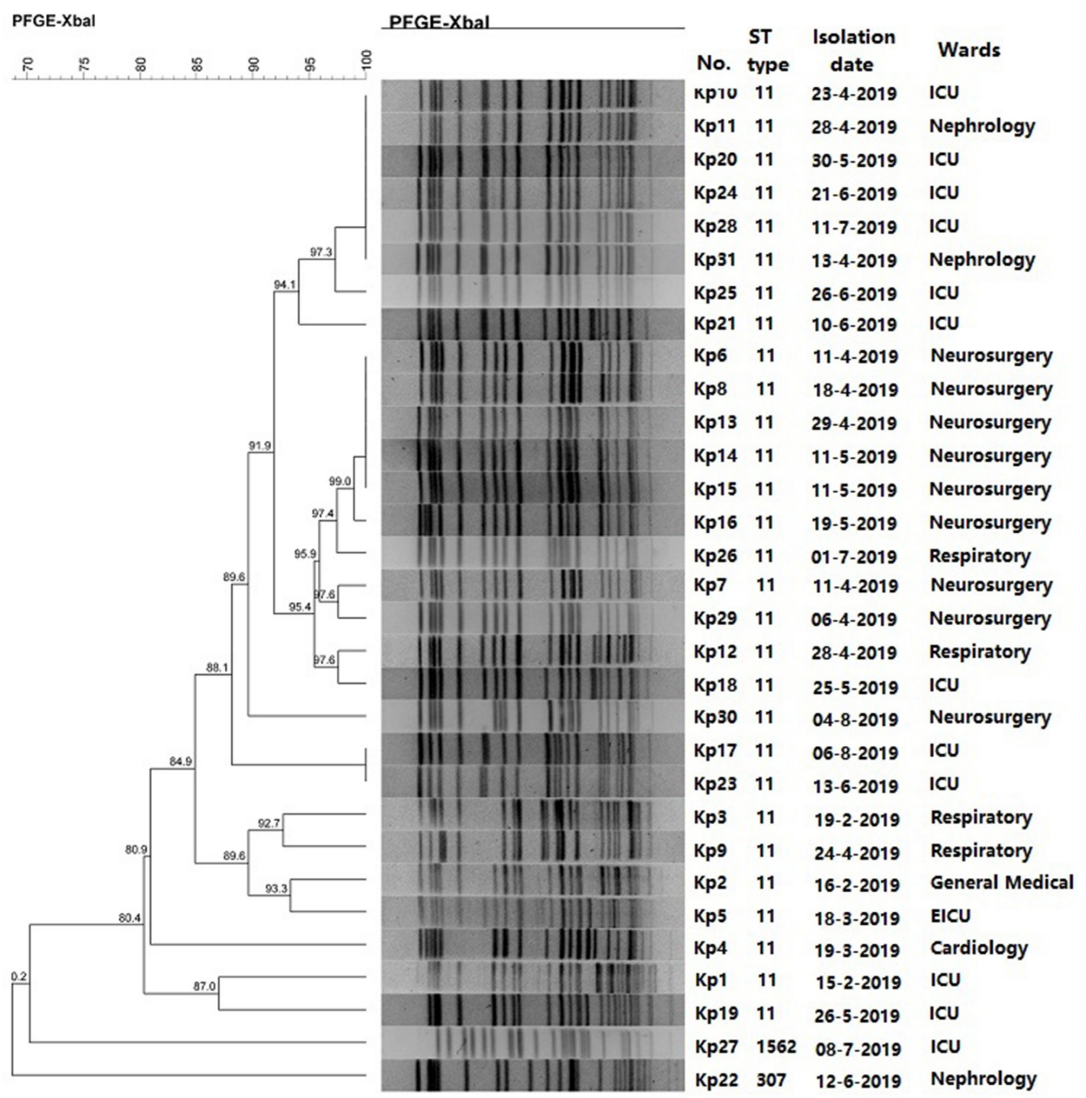
PFGE results and MLST typing for 31 CRKp isolates.

MLST analysis revealed that 29 CRKps carrying *bla*_KPC−2_ were ST11 type, which was the most common type of CRKp found in China. Moreover, *bla*_NDM−5_ carrying Kp22 and Kp27 was assigned to ST307 and ST1562, respectively. For PFGE, a cluster was defined as strains with homology >80%. The results revealed that the homology of 29 isolates of ST11 was more than 80%, indicating a cluster; however, no homology between the two strains carrying *bla*_NDM−5_ (Figure 1). Notably, the eight CRKps from neurosurgery, between April 6, 2019 and May 19, 2019, showed high homology (≥95%).

### Conjugation and Plasmid Analysis

**Figure 2:**
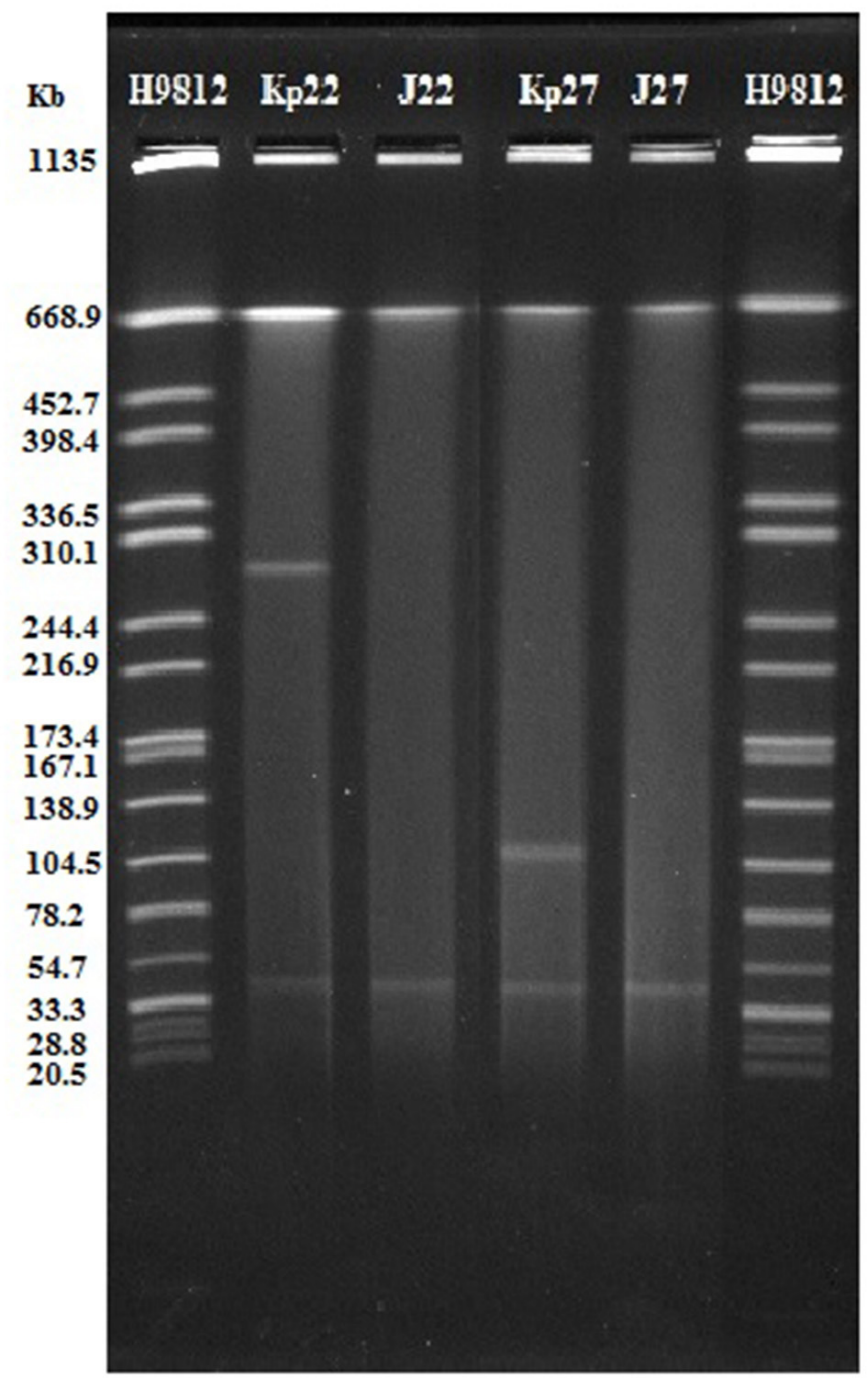
**S1-**PFGE results for Kp22 and Kp27 and their transconjugants.

Our results revealed that *bla*_NDM−5_ was successfully transferred to *E. coli J53* from Kp22 and Kp27 isolates. The corresponding transconjugants are termed J22 and J27, respectively. Regrettably, the *bla*_KPC−2_ gene failed to be transferred. Compared to the recipient strain *E. coli J53*, J22, and J27 exhibited significantly reduced carbapenem susceptibility (Table 2). The sensitivities of cephalosporin, β-lactam/β-lactamase inhibitor combinations, and carbapenems were similar to those of the donor strains, and the sensitivity of J22 and J27 to aztreonam, trimethoprim-sulfamethoxazole, quinolones, aminoglycosamines, tigecycline, and colistin were similar to those of *E. coli J53*. Moreover, S1-PFGE revealed that 31 CRKp isolates carried 1–4 plasmids (Supplementary Figure 1). Both Kp22 and Kp27 contained two plasmids. After conjugation, J22 and J27 contained only one plasmid, about 45 kb in size (Figure 2). Furthermore, PCR-based replicon typing revealed that these two plasmids belong to the IncX3 incompatibility group.

## Discussion

Respiratory tract infections can occur at any age; however, LRTIs is more common in the elderly people. Moreover, pneumonia is a leading cause of illness and death in the elderly (13). Viruses are responsible for a large proportion of LRTIs but antibiotics are often unnecessarily prescribed for their treatment without any laboratory testing and can contribute to the emergence of antimicrobial resistance (14). *K. pneumoniae* is the predominant bacterial pathogen of LRTIs, and the positive rate of CRKp is increasing year by year, particularly for elderly patients (15, 16). Analysis of the molecular characteristics and drug resistance mechanism of CRKp from elderly patients with LRTIs can prove beneficial in treating this kind of infection.

In China, KPC-2 is the predominant carbapenemase in CRKp, and ST11 is the major sequence type of CRKp (17, 18). Similarly, the majority (93.55%) of CRKp isolates in the present study were KPC-2-producing ST11. We isolated two strains of *bla*_NDM−5_ carrying *K. pneumoniae* in the present study. In addition to *bla*KPC-2 and *bla*_NDM−5_, all the 31 isolates harbored *bla*TEM-1. Moreover, majority of these isolates carried *bla*_SHV−11_ and *bla*_CTX−M−1_, which was in accordance to the results of the previous reports (19, 20). These resistance genes led to the resistance of 31 isolates to β-lactams. Furthermore, most isolated strains were resistant to aminoglycosides and quinolones, which made the clinical fewer antibiotics choice. Fortunately, all strains were sensitive to tigecycline and colistin.

The emergence and spread of New Delhi metallolactamase (NDM)-producing *Enterobacteriaceae* has posed a serious public health concerns. The NDM-5 carbapenemase differs from NDM-1 by only two amino acid substitutions (Val88Leu and Met154Leu) and exhibits increased resistance to carbapenems and expanded-spectrum cephalosporins (21). NDM-5 has been identified mostly in *E. coli* but has rarely been described in *K. pneumoniae* and other *Enterobacteriaceae* isolates. In the present study, two *K. pneumoniae* isolates carrying *bla*_NDM−5_ came from different wards. They were assigned to be ST307 and ST1562. NDM-5 carried ST307 *K. pneumoniae* has been reported previously (21, 22). To the best of our knowledge, this is the first report of ST 1562 types with *bla*_NDM−5_. The *bla*_NDM−5_ gene was located on a conjugative IncX3 plasmid. In 2019, Ziyan Kong reported a nosocomial outbreak of NDM-5-producing *K. pneumoniae* in a neonatal unit in China (8). They demonstrated that all *bla*_NDM−5_ genes were located on a ~45 kb IncX3 type plasmid. The study of Zhu et al. (21) showed that the IncX3 plasmid facilitated the dissemination of *bla*_NDM−5_ among multiclonal *K. pneumoniae* strains and that conjugal transfer contributed significantly to IncX3 plasmid stability within *K. pneumoniae*. Hence, there is an urgent need for effective infection control measures to prevent *bla*_NDM−5_ variants from causing epidemic in the future.

Over the past decade, carbapenem-resistant hypervirulent *K. pneumoniae* (CR-hvKP) has gained immense attention (23). In this study, the serotypes closely related to hypervirulent *K. pneumoniae* and 12 virulence-associated genes of 31 CRKp isolates were analyzed. Six virulence-associated genes, *fim, uge, mrkD, wabG, rmpA*, and *iucB* were harbored by almost 31 isolates. These virulence genes are related to capsule synthesis, flagella movement, and iron acquisition, which are virulence factors of *K. pneumonia* (24). Fortunately, several serotypes of hypervirulent *K. pneumoniae* remained undetected. Eventually, 31 patients revealed good prognosis, and only one patient died.

The main risk factors of CRKp infection include immunosuppression, ICU admission, antibiotics exposure, surgery, mechanical ventilation, and central venous catheterization (25, 26). Elderly patients with LRTI often present non-specific symptoms, which are often covered by primary diseases, leading to irrational use of antibiotics (27). In the present study, 29 patients were exposed to various antimicrobial drugs before the positive culture. Piperacillin/tazobactam was the most commonly used antibiotic in 31 patients. Based on *in vitro* susceptibility testing results, majority of these patients changed to appropriate drugs such as tigecycline, levofloxacin, and amikacin. Importantly, nervous system diseases including intracranial hemorrhage and injury are the most common underlying diseases in this study. Most of these patients are unconscious and were subjected to mechanical ventilation. This is in accordance with the results of previous studies (26). Admission to neurosurgery is also a risk factor for CRKp infection. In addition, CRKp strains from neurosurgical patients have high homology, which indicates that there may be an outbreak of nosocomial infection of CRKps in neurosurgery; however, as this study was retrospective, we could not perform in-depth analysis of the patient's surrounding environment and the source of the strain.

In summary, we reported the characteristics of CRKp isolates collected from elderly patients with LRTIs. The high incidence of CRKp highlights the urgent need for further surveillance and strict infection control measures, particularly for ICU patients and immune-compromised elderly patients. One *bla*_NDM−5_ carrying CRKp with ST1562 were first reported.

## Data Availability Statement

The original contributions presented in the study are included in the article/Supplementary Material, further inquiries can be directed to the corresponding author/s.

## Ethics Statement

The studies involving human participants were reviewed and approved by the Ethics Committee of Taian City Central Hospital. Written informed consent for participation was not required for this study in accordance with the national legislation and the institutional requirements.

## Author Contributions

MJ and FZ designed the experiments and revised the manuscript. CS carried out the experiments and wrote the manuscript. WW and SL analyzed the data. ZZ contributed to experiment conception. All authors contributed to the article and approved the submitted version.

## Conflict of Interest

The authors declare that the research was conducted in the absence of any commercial or financial relationships that could be construed as a potential conflict of interest.

## Supplementary Material

Supplementary Figure 1 S1-PFGE results for 31 CRKp isolates.

Supplementary Table 1 Primers used in the present study.
