# Supplementary figures and images for: Molecular Epidemiology and Drug Resistant Mechanism of Carbapenem-Resistant Klebsiella pneumoniae in Elderly Patients With Lower Respiratory Tract Infection
Source: Front Public Health. 2021 May 20;9:669173. doi: 10.3389/fpubh.2021.669173 (PMC8172620; doi:10.3389/fpubh.2021.669173)

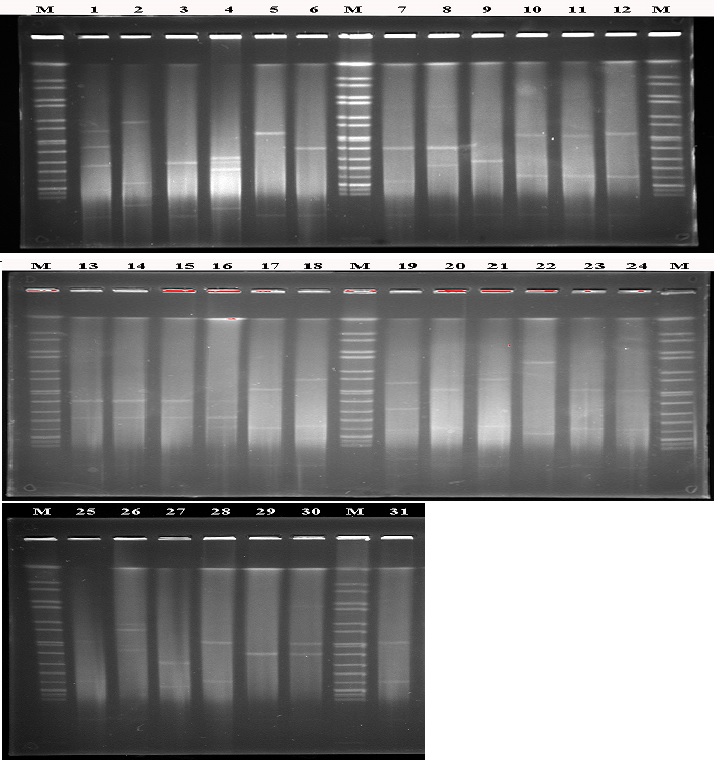

Supplement: Supplementary Figure 1 — S1-PFGE results for 31 CRKp isolates. [file Image_1.JPEG]
