# Supplementary material for: Molecular Epidemiology and Drug Resistant Mechanism of Carbapenem-Resistant Klebsiella pneumoniae in Elderly Patients With Lower Respiratory Tract Infection
Source: Front Public Health. 2021 May 20;9:669173. doi: 10.3389/fpubh.2021.669173 (PMC8172620; doi:10.3389/fpubh.2021.669173)
Supplement: Supplementary Table 1 — Primers used in the present study. [file Table_1.doc]

**Supplementary table 1 Primers used in the present study**

| Primer | Forward (5'-3') | Reverse (5'-3') |
| --- | --- | --- |
| *bla*_KPc_ | ATTGGCTAAAGGGAAACACGACC | GTAGACGGCCAACACAAT |
| *bla*_SME_ | AGATAGTAAATTTTATAG | CTCTAACGCTAATAG |
| *bla*_IMI_ | ATAGCCATCCTTGTTTAGCTC | TCTGCGATTACTTTATCCTC |
| *bla*_NMC_ | GCATTGATATACCTTTAGCAGAGA | CGGTGATAAAATCACACTGAGCATA |
| *bla*_GES_ | GTTTTGCAATGTGCTCAACG | TGCCATAGCAATAGGCGTAG |
| *bla*_IMP_ | TGAGCAAGTTATCTGTATTC | TTAGTTGCTTGGTTTTGATG |
| *bla*_VIM_ | TTATGGAGCAGCAACCGATGT | CAAAAGTCCCGCTCCAACGA |
| *bla*_GIM_ | AGAACCTTGACCGAACGCAG | ACTCATGACTCCTCACGAGG |
| *bla*_SIM_ | TACAAGGGATTCGGCATCG | TAATGGCCTGTTCCCATGTG |
| *bla*_SPM_ | CCTACAATCTAACGGCGACC | TCGCCGTGTCCAGGTATAAC |
| *bla*_NDM_ | ATTAGCCGCTGCATTGAT | GGCATGTCGAGATAGGAAGT |
| *bla*_OXA-48 like_ | ACACAATACATATCAACTTCGC | AGTGTGTTTAGAATGGTGATC |
| *bla*_CTX-M-1_ | GGTTAAAAAATCACTGCGTC | TTACAAACCGTYGGTGACGA |
| *bla*_CTX-M-2_ | ATGATGACTCAGAGCATTCGCCGC | TCAGAAACCGTGGGTTACGATTTT |
| *bla*_CTX-M-9_ | GTGACAAAGAGAGTGCAACGG | ATGATTCTCGCCGCTGAAGCC |
| *bla*_CTX-M-15_ | CACACGTGGAATTTAGGGACT | GCCGTCTAAGGCGATAAACA |
| *bla*_TEM_ | ACATGGGGGATCATGTAACT | GACAGTTACAATGCTTACT |
| *bla*_SHV_ | ATGCGTTATATTCGCCTGTG | AGCGTTGCCAGTGCTCGATG |
| *bla*_MOX_ | GCTGCTCAAGGAGCACAGGAT | CACATTGACATAGGTGTGGTGC |
| *bla*_FOX_ | AACATGGGGTATCAGGGAGATG | CAAAGCGCGTAACCGGATTGG |
| *bla*_DHA_ | AACTTTCACAGGTGTGCTGGGT | CCGTACGCATACTGGCTTTGC |
| *bla*_CIT_ | TGGCCAGAACTGACAGGCAAA | TTTCTCCTGAACGTGGCTGGC |
| *bla*_EBC_ | TCGGTAAAGCCGATGTTGCGG | CTTCCACTGCGGCTGCCAGTT |
| Int1 | CCTCCCGCACGATGATC | TCCACGCATCGTCAGGC |
| Int2 | TTATTGCTGGGATTAGGC | ACGGCTACCCTCTGTTATC |
| Int3 | AGTGGGTGGCGAATGAGTG | TGTTCTTGTATCGGCAGGTG |
| *qnrA* | ATTTCTCACGCCAGGATTTG | GATCGGCAAAGGTTAGGTCA |
| *qnrB* | GATCGTGAAAGCCAGAAAGG | ACGATGCCTGGTAGTTGTCC |
| *qnrC* | GGGTTGTACATTTATTGAATCG | CACCTACCCATTTATTTTCA |
| *qnrD* | GGGTTGATTTAACTGATAC | TTCGCACTTTTCTAATATGAC |
| *qnrS* | ACGACATTCGTCAACTGCAA | TAAATTGGCACCCTGTAGGC |
| *qepA* | GCAGGTCCAGCAGCGGGTAG | CTTCCTGCCCGAGTATCGTG |
| *aac(6)-Ib-cr* | TTGCGATGCTCTATGAGTGGCTA | CTCGAATGCCTGGCGTGTTT |
| *rmtA* | AAACTATTCCGCATGGTTC | TCATGTACACAAGCTCTTTCC |
| *rmtB* | ACTTTTACAATCCCTCAATAC | AAGTATATAAGTTCTGTTCCG |
| *rmtC* | CAGGGGTTCCAACAAGT | AGAGTATATAGCTTGAACATAAGTAGA |
| *rmtD* | GGAAAAGGACGTGGACA | TCCATCGATTCCACAGG |
| *npmA* | GGGCTATCTAATGTGGTG | TTTTTATTTCCGCTTCTTCGT |
| *armA* | ATTTTAGATTTTGGTTGTGGC | ATCTCAGCTCTATCAATATCG |
| K1 | GGTGCTCTTTACATCATTGC | GCAATGGCCATTTGCGTTAG |
| K2 | GACCCGATATTCATACTTGACAGAG | CCTGAAGTAAAATCGTAAATAGATGGC |
| K5 | TGGTAGTGATGCTCGCGA | CCTGAACCCACCCCAATC |
| K20 | CGGTGCTACAGTGCATCATT | GTTATACGATGCTCAGTCGC |
| K54 | CATTAGCTCAGTGGTTGGCT | GCTTGACAAACACCATAGCAG |
| K57 | CTCAGGGCTAGAAGTGTCAT | CACTAACCCAGAAAGTCGAG |
| *rmpA* | ACTGGGCTACCTCTGCTTCA | CTTGCATGAGCCATCTTTCA |
| *aerobactin* | GCATAGGCGGATACGAACAT | CACAGGGCAATTGCTTACCT |
| *wcaG* | GGTTGGKTCAGCAATCGTA | ACTATTCCGCCAACTTTTGC |
| *ybtA* | ATGACGGAGTCACCGCAAAC | TTACATCACGCGTTTAAAGG |
| *iucB* | ATGTCTAAGGCAAACATCGT | TTACAGACCGACCTCCGTGA |
| *iroNB* | GGCTACTGATACTTGACTATTC | CAGGATACAATAGCCCATAG |
| *ureA* | GCTGACTTAAGAGAACGTTATG | GAAGATCAGTCACACCATCC |
| *uge* | GATCATCCGGTCTCCCTGTA | TCTTCACGCCTTCCTTCACT |
| *kfuBC* | GAAGTGACGCTGTTTCTGGC | TTTCGTGTGGCCAGTGACTC |
| *fim* | GCTCTGGCCGATACTACCACGG | GCGAAGTAACGCGCCTGGAACGG |
| *wabG* | CGGACTGGCAGATCCATATC | ACCATCGGCCATTTGATAGA |
| *allS* | CCGTTAGGCAATCCAGAC | TCTGATTTATCCCACATT |
